# False-Positive Screening and Confirmatory HIV Diagnostic Test in a Patient with Cured SARS-CoV-2 Infection Is Not Mediated by Env/Spike Cross-Reactive Antibodies

**DOI:** 10.3390/v15051161

**Published:** 2023-05-13

**Authors:** Carina Elsner, Gwenllian A. Appeltrath, Margarethe Konik, Janine Parreuter, Martina Broecker-Preuss, Adalbert Krawczyk, Stefan Esser, Stefanie Sammet, Christina B. Karsten

**Affiliations:** 1 Institute for Virology, University Hospital Essen, University of Duisburg-Essen, 45147 Essen, Germany; 2 Institute for Translational HIV Research, University Hospital Essen, University of Duisburg-Essen, 45147 Essen, Germany; 3 Department of Infectious Diseases, West German Centre of Infectious Diseases, University Hospital Essen, University Duisburg-Essen, 45147 Essen, Germany; 4 Laboratory Medicine Section, Department of Medicine, University Hospital Knappschaftskrankenhaus Bochum, Ruhr University Bochum, 44892 Bochum, Germany; 5 Clinic of Dermatology, University Hospital Essen, University of Duisburg-Essen, 45147 Essen, Germany

**Keywords:** HIV-1, SARS-CoV-2, cross-reactive antibodies, diagnostic test

## Abstract

Acute SARS-CoV-2 infection has been associated with false-positive HIV screening tests. The underlying mechanism is unclear, and for clinical cases, evidence beyond a temporal connection is missing. However, several experimental studies point toward SARS-CoV-2 spike/HIV-1 envelope (Env) cross-reactive antibodies (Abs) as a cause. Here, we present the first case of an individual with convalescent SARS-CoV-2 infection testing false positive in both an HIV screening and confirmatory test. Longitudinal sampling showed that the phenomenon was temporary but lasted for at least 3 months before waning. After excluding a multitude of common determinants for assay interference, we further show by antibody depletion studies that SARS-CoV-2-spike-specific Abs did not cross-react with HIV-1 gp120 in the patient sample. No additional case of HIV test interference was identified in a cohort of 66 individuals who presented to a post-COVID-19 outpatient clinic. We conclude the SARS-CoV-2-associated HIV test interference to be a temporary process capable of disturbing both screening and confirmatory assays. The assay interference is short-lived and/or rare but should be considered by physicians as a possible explanation for unexpected HIV diagnostic results in patients with a recent SARS-CoV-2 infection.

## 1. Introduction

Highly sensitive and specific diagnostic assays are important for identifying an infectious disease. False-positive test results are rare, but especially in the case of a screening test to examine an infection with the human immunodeficiency virus (HIV), the causative agent for the development of the acquired immunodeficiency syndrome (AIDS), a positive result has substantial emotional and social consequences for the patient. To minimize the risk of a false-positive HIV diagnosis, a reactive HIV antigen/antibody (Ab) screening test has to be confirmed by either an immunoblot and/or a quantitative PCR test for HIV RNA, and a suspected diagnosis should be confirmed using an independent sample [1].

A variety of conditions can interfere with HIV diagnostic assessments. These include high concentrations of biotin often used as a dietary supplement, which interfere with streptavidin–biotin interactions in detection systems [2], as well as pregnancy [3], autoimmune disease [4], cancer [5], vaccination against influenza [6] or rubella virus [7], and receipt of blood donations [8]. Furthermore, infections with different protozoa, bacteria, and viruses, including Epstein–Barr virus (EBV) [9], hepatitis A [10] and B [11], measles [12], and dengue virus [13] have been linked to HIV diagnostic malperformance. 

In addition to the previously recognized interfering reagents, three cohort studies found that coronavirus disease (COVID-19) was significantly associated with false-positive results in the fourth-generation HIV screening tests Elecsys HIV Duo (Roche) [14,15] and Genscreen Ultra HIV Ag-Ab (Bio-Rad) [16]. Further, severe acute respiratory syndrome coronavirus type 2 (SARS-CoV-2)-associated HIV test reactivity was linked in case studies to additional HIV screening tests including Elecsys HIV Combi PT (Roche) [17], VIDAS HIV Duo Ultra (BioMérieux) [18], Vitros HIV combo (Ortho Clinical Diagnostics) [19], Architect HIV Ag/Ab Combo (Abbott) [18,20,21], or unspecified tests [22,23]. In all cases, confirmatory immunoblots for HIV Abs and/or qPCRs for HIV RNA were negative. Conversely, false-positive results were also observed in the SARS-CoV-2 antigen test Espline SARS-CoV-2 (Fujirebio) during acute HIV infection [24]. 

Although striking differences exist in the biology of SARS-CoV-2 and HIV, their proteins for cell attachment share common features. Both the SARS-CoV-2 spike and HIV envelope (Env) are class 1 fusion machineries, require furin cleavage to generate their surface (S1/gp120) and transmembrane (S2/gp41) subunits, and are heavily glycosylated [25]. Based on these structural similarities, several authors proposed SARS-CoV-2 spike/HIV Env cross-reactive Abs as an explanation for the observed false-reactive HIV screening tests post SARS-CoV-2 infection [15,17,18,19,20]. No attempt has yet been made to demonstrate the existence of this type of Abs in patient sera with HIV test interference; however, vaccination of mice with spike-induced Abs capable of binding the HIV gp41 membrane-proximal external region (MPER) suggests that induction of HIV cross-reactive Abs by SARS-CoV-2 is possible [26]. Furthermore, broadly neutralizing Abs (bnAbs) induced by HIV recognizing MPER were found to bind efficiently to SARS-CoV-2 spike [27]. Moreover, bnAbs targeting partially or fully glycan-dependent epitopes on the HIV envelope subunit gp120 bind spike [28,29]. In summary, in the absence of direct proof of SARS-CoV-2-induced HIV cross-reactive Abs in human clinical samples, animal and in vitro studies support cross-reactive Abs as a possible cause for HIV diagnostic test interference.

Here, we present an investigative case study describing false-positive HIV diagnostic test results following a cured SARS-CoV-2 infection that was observed for the first time in both an HIV screening and confirmatory test. Further, we demonstrate that SARS-CoV-2 spike/HIV-1 Env cross-reactive Abs were not responsible for this several months long lasting assay interference.

## 2. Materials and Methods

### 2.1. Clinical Samples and Data

The individual with suspected HIV diagnosis due to a reactive HIV screening and confirmatory test (patient 1) whose case is described here was recruited for the study during an appointment at the HPSTD-outpatient center of the University Hospital Essen. The participants of the post-COVID cohort were included retrospectively or actively recruited during their appointments for treatment at the post-COVID outpatient center of the University of Duisburg-Essen. Clinical data were obtained through historical chart review or interview of the participants.

### 2.2. Certified Diagnostic Tests

Blood counts were determined at the Central Laboratory of the University Hospital Essen according to routine procedures. For HIV-1/2 diagnostics, the screening tests Architect HIV Ag/Ab Combo (chemiluminescent microparticle immunoassay (CMIA); Abbott, Wiesbaden, Germany) and Elecsys HIV combi PT (electrochemiluminescence immunoassay; Roche Diagnostics, Rotkreuz, Switzerland) were utilized. Further, the confirmatory test INNO-LIA HIV I/II Score (line-immunoassay; Fujirebio, Hannover, Germany) and RealTime HIV-1 m2000sp (qPCR; Abbott, Wiesbaden, Germany) were employed. The presence of an EBV infection was assessed using the chemiluminescence immunoassays (CLIAs) Liaison EBV IgM, VCA IgG, and EBNA IgG (all DiaSorin, Dietzenbach, Germany). SARS-CoV-2-specific Abs were measured using the SARS-CoV-2 S1/S2 IgG ELISA (DiaSorin) and the CLIA Liaison SARS-CoV-2 TrimericS IgG assay (DiaSorin) for spike-specific Abs (IgG S), while the CMIA SARS-CoV-2 IgG assay (Abbott) was used for nucleocapsid (N)-specific Abs (IgG N).

### 2.3. Protein Production

For the production of a soluble trimeric SARS-CoV-2 spike protein, a total of 250 µg of plasmid DNA (pCAGGS-soluble trimeric HIS-tagged SARS-CoV-2 spike protein, strain Wuhan-Hu-1 [30]) and 750 µL of PEI at a concentration of 1 mg/mL in ddH_2_O was diluted separately in 12.5 mL of Opti-MEM (Thermo Fisher Scientific, Dreieich, Germany), sterile filtered, and combined after a 5 min incubation period at room temperature. After 30 min of incubation, the transfection mixture was added dropwise to a 500 mL culture of Freestyle 293-F cells in FreeStyle 293 Expression Medium (Thermo Fisher Scientific) at a density of 1.2 × 10^6^ cells/mL. The cells were incubated at 37 °C, 8% CO_2_, and 70% humidity on an orbital shaker platform rotating at 135 rpm. After five days, the cells were centrifuged (2470× *g*, 20 min) and the supernatant was filtered using a 0.8/0.2 µm VacuCap filtration device (Pall, Crailsheim, Germany). Subsequently, the supernatant was buffer exchanged into PBS using a tangential flow filtration system (Pall) and incubated with 2.5 mL of Ni Sepharose 6 Fast Flow (Cytiva, Freiburg im Breisgau, Germany) overnight at 4 °C on a shaking table. The next day, the beads were collected in an Econo-column (Bio-Rad, Feldkirchen, Germany) prewetted with PBS. The resin was washed with 100 mL of PBS and 50 mL of 20 mM imidazole in PBS, and the proteins were eluted using 80 mL of 500 mM imidazole in PBS. The protein elution was concentrated in Amicon Ultra Centrifugal Filters (3 k cutoff; Merck, Darmstadt, Germany) at 3010× *g*. Proteins were further purified with size exclusion chromatography using a Superdex 200 Increase 10/300 GL column with PBS as the eluent on an Äkta pure 25L FPLC (both Cytiva).

### 2.4. Antigen-Specific Ab Depletion

To prepare the streptavidin-coated magnetic beads (Acro-Biosystems, Newark, DE, USA) for Ab depletion of serum samples, the beads were reconstituted in ddH_2_O to 1 mg/mL. Next, beads were washed three times through the addition of 1 mL of assay buffer (0.05% BSA in PBS (PBSA) with 0.05% Tween-20), vortexing was applied for 1 min, the tube was placed in a magnet, and the supernatants were removed. Washed beads were diluted to 1 mg/mL in assay buffer and stored at −20 °C until use. The antigen-soluble SARS-CoV-2 spike protein, HIV-1 gp120 clade B’/C consensus (Immune Technology, New York, NY, USA) or BSA (used for mock depletions, Roche) was biotinylated via incubation at room temperature for 30 min with a 20-fold molar excess of EZ-Link Sulfo NHS-LC-LC-Biotin (Thermo Fisher Scientific). Excess biotin was removed using Zeba desalting columns (Thermo Fisher Scientific). The columns were prepared by 4 washes with 300 µL of PBS (1 min, 1500× *g*). Subsequently, the biotinylated antigen was added and moved through the matrix via a 2 min centrifugation for 1500× *g*. For the depletion of the Abs of serum samples, serum and biotinylated antigen (ratio 5:1, µL/µg) were combined in a 96-well cell culture plate. The plate was sealed with parafilm and incubated overnight under shaking at 4 °C. After the removal of supernatant from beads, the serum–antigen mix was added to the beads (ratio 2:1), and the reaction vortexed for 1 min. All was transferred to a 96-well cell culture plate, which was sealed with parafilm and incubated under shaking for 1 h. The mixture was placed in a magnetic field for 2 min, and the Ab-depleted supernatants were collected for immediate analysis.

### 2.5. Enzyme-Linked Immunosorbent Assays (ELISAs)

Ab responses to the Epstein–Barr virus (EBV) early antigen (EA) were analyzed using the Epstein–Barr virus EA IgG ELISA (Tecan, Crailsheim, Germany) according to the manufacturer’s guidelines. For the measurement of SARS-CoV-2 spike-specific IgM or IgG, 96-well Nunc MaxiSorp ELISA plates (Thermo Fisher Scientific) were coated with 100 µL per well of SARS-CoV-2 spike (5 µg/mL) or HIV-1 gp120 clade B’/C consensus (1 µg/mL, Immune Technology) in 0.1 M NaHCO_3_ (pH 8.6) and incubated at 4 °C overnight. The plates were washed four times with 400 µL of PBS with 0.05% Tween-20, blocked with 360 µL of PBSA (5% BSA in PBS)/0.01% Tween-20 for 1 h, and washed again four times. Subsequently, 100 µL of sample diluted in PBSA with 20% sheep serum was added per well and incubated for 2 h. The plates were washed six times and 100 µL of HRP-coupled mouse anti-human IgM heavy chain (1:4000; Invitrogen, Karlsruhe, Germany) or goat anti-human IgG Fc (1:10,000; Sigma-Aldrich, Taufkirchen, Germany) in PBSA was added per well. After a 1 h incubation, the plates were washed six times, 100 µL of 1-step Ultra TMB-ELISA substrate solution (Thermo Fisher Scientific) was added to the wells, and the plate was incubated in the dark for 30 min. The reaction was stopped by the addition of 100 µL of 1 N sulfuric acid to each well, and the absorbance was read at 450 nm with 570 nm reference wavelengths on a Spark Reader (Tecan). Graphs were plotted using GraphPad Prism (version 9.5.0., GraphPad Software).

## 3. Case Description

A 32-year old woman (patient 1) was referred to the HIV outpatient center at the University Hospital Essen with a reactive HIV screening test and a positive confirmatory test result during routine diagnostics after plasma donation in December 2020 suggesting a possible HIV-1 infection. At her visit on-site in the same month, the patient reported that she donates plasma regularly and there have been no abnormalities in her laboratory values thus far (last plasma donation without laboratory abnormalities June 2020). The patient experienced a mild SARS-CoV-2 infection as confirmed with qPCR in October 2020 and additionally with CMIA (SARS-CoV-2 IgG assay (Abbott)) in December 2020; the patient had no medical history and was not taking any medication or dietary supplements. The blood count was unremarkable except for slightly increased liver values (ALT 45 U/L, AP 120 U/L, and GGT 53 U/L). The initial fourth-generation HIV screening test (Elecsys HIV combi PT, Roche) was repeatedly reactive (COI values 2.45, 2.60, and 2.56), and the confirmatory immunoblot (INNO-LIA HIV I/II Score, Fujirebio) was positive for gp120 (2+) and gp41 (2+). HIV-1 qPCR was negative according to the external laboratory report.

## 4. Results

### 4.1. The HIV Diagnostic Test Interference after Acute SARS-CoV-2 Infection Resolved over Time

**Figure 1:**
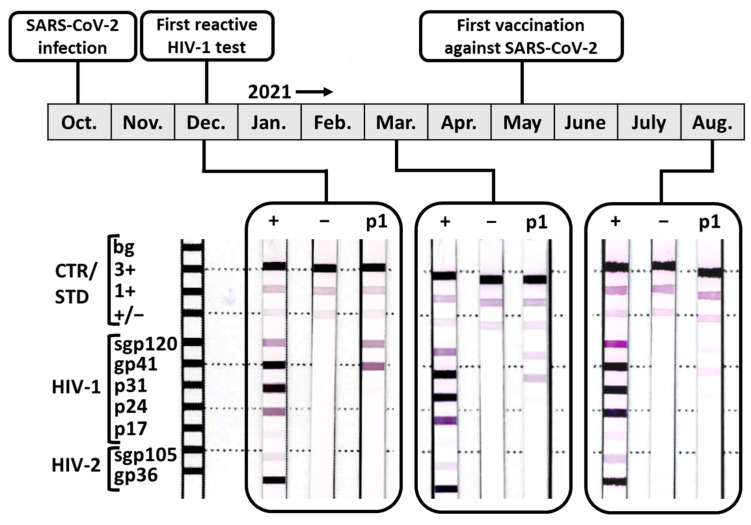
Timeline for patient 1 including scans of the results of her confirmatory HIV diagnostic test (INNO-LIA HIV I/II Score, Fujirebio). +: positive control; −: negative control; p1: patient 1.

We retested patient 1 at the University Hospital Essen twelve days later using a different fourth-generation HIV screening test (Architect HIV Ag/Ab Combo, Abbott). While this assay was negative (0.21 S/CO), confirmatory testing using the identical immunoblot used previously (INNO-LIA HIV I/II Score, Fujirebio) showed sample reactivity (HIV-1 gp120 (2+) and gp41 (2+), Figure 1). Again, no viral RNA was detectable by HIV-1 qPCR (Abbott RealTime HIV-1 m2000sp, Abbott). To determine the persistence of the assay interfering substances, patient 1 was asked to donate again eleven weeks later in March 2021, and the HIV screening and confirmatory test was repeated (Figure 1). Both screening tests (Architect HIV Ag/Ab Combo, Abbott and Elecsys HIV combi PT, Roche) were negative (0.26 S/CO; COI 0.145), and the intensity of the immunoblot reaction was diminishing, resulting in a questionable result (HIV-1 gp120 +1 and gp41 +1). A third sample was collected in August 2021 showing that both HIV screening tests (Architect HIV Ag/Ab Combo, Abbott; Elecsys HIV combi PT, Roche) and the confirmatory immunoblot were negative (0.27 S/CO; COI 0.166; HIV-1 gp120 < ± and gp41 < ±) (Figure 1). These findings suggest that the substance interfering with the HIV diagnostic ELISA and immunoblots was waning over time, and therefore a temporary effect was observed.

Although reports of SARS-CoV-2-linked HIV test reactivity are accumulating, instances of this phenomenon might be missed since HIV tests are usually not performed as a means for differential diagnosis to COVID-19 or post-COVID. Due to our work at the specialist center of care for long-COVID patients at the University Hospital Essen, we had the opportunity to investigate a cohort of 66 individuals for HIV test reactivity post SARS-CoV-2 infection (Table 1). Our participants were 64.6% female and 35.4% male and had a median age (IQR) of 51 (19). Their SARS-CoV-2 infection was documented with qPCR a median (IQR) of 9 months (5) prior to the biospecimen collection. Further, at the time of sampling, 81.5% of individuals were vaccinated, 6.2% were not, and 12.3% had an unknown vaccination status. A SARS-CoV-2-specific IgG response against the spike protein was detected in 96.9% of serum donors using the Liaison SARS-CoV-2 TrimericS IgG assay (DiaSorin). Additionally, the IgG response to the SARS-CoV-2 N protein was assessed using the CMIA SARS-CoV-2 IgG assay (Abbott). Overall, 41.5% of the cohort participants tested positive for N protein-specific IgG, 13.8% were borderline reactive, and 44.6% of the samples showed no response. None of the donors was reactive in HIV-1/2 screening (Elecsys HIV combi PT (Roche), Architect HIV Ag/Ab Combo test, Abbott), or confirmatory tests (INNO-LIA HIV I/II Score, Fujirebio). Based on the findings generated from this small cohort, we conclude that SARS-CoV-2-induced HIV test reactivity is most likely a rather rare event and/or so short-lived that it is not detectable in a cohort of individuals sampled about 9 months past their SARS-CoV-2 infection.

### 4.2. The Assessment of Common Types of Interferences Could Not Disprove a Potential Relationship between SARS-CoV-2 Infection and Subsequent False-Positive HIV Diagnostic Tests

To exclude the presence of substances known to interfere with diagnostic immunoassays, patient 1 was asked to recall a past EBV infection or any other relevant health events in the months before her plasma donation, including any pregnancies, long-term exposures to animals, chronic diseases, nicotine consumption, treatments with blood or blood products, infections, or vaccinations. Apart from reporting one live birth two years prior, patient 1 could not recall any relevant information in this regard. Human antianimal Abs could be excluded since the Abs utilized in the false-positive HIV tests of patient 1 were derived from different animal sources according to the manufacturers. 

**Figure 2:**
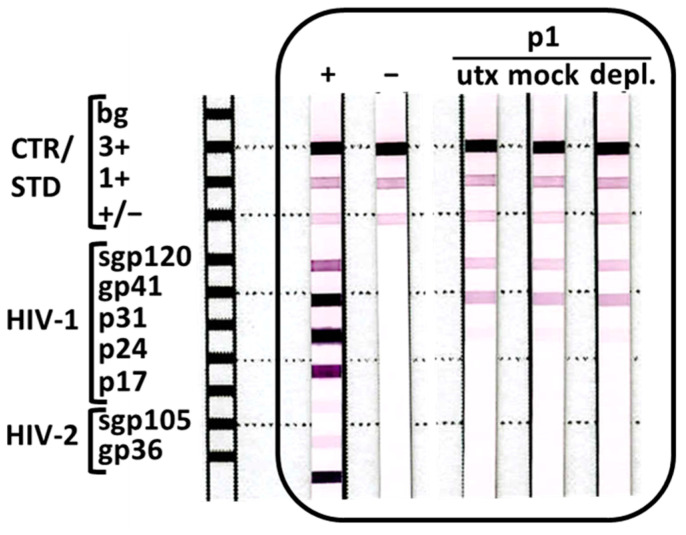
Depletion of potentially present streptavidin-specific Abs in the serum of patient 1. The serum of patient 1 (p1) collected in March 2021 was analyzed using the INNO-LIA HIV I/II Score assay either untreated (utx), mock depleted using BSA-covered commercially available streptavidin beads, or depleted for streptavidin-specific Abs (depl.) using untreated streptavidin beads; shown is an annotated scan of the results; n = 1.

Streptavidin-specific Abs are listed as a known source of interference for the INNO-LIA HIV I/II Score assay in the assay handbook. According to the manufacturer, the presence of interfering antistreptavidin Abs is likely to occur when the bands for HIV-1 gp120 and gp41 are solely reactive, as was observed for patient 1, independent from a nonresponsive background control. Thus, to confirm the absence of interfering streptavidin Abs, a serum sample of patient 1 collected in March 2021 was either mock treated or depleted with streptavidin-coated beads with or without biotinylated BSA before the INNO-LIA HIV I/II Score assay was repeated (Figure 2). The depletion of potential Abs with streptavidin reactivity had no effect on the HIV diagnostic test. Since active EBV infection can produce heterophil Abs interfering with diagnostic tests [9], we characterized the EBV status of patient 1 in March 2021. Serological testing confirmed a past EBV infection (VCA-IgM^−^ (<10 U/mL), VCA-IgG^+^ (459 U/mL), and EBNA1-IgG^+^ (>600 U/mL) (Liaison EBV IgM, VCA IgG, and EBNA IgG, DiaSorin) with no virus reactivation (<8 U/mL; EBV EA IgG ELISA, Tecan). Thus, we concluded that the temporary HIV test reactivity of patient 1 was caused by a none of these common interfering substances.

### 4.3. Cross-Reactive Abs to SARS-CoV-2 Spike and HIV-1 gp120 Did Not Cause HIV Diagnostic Test Interference

The work of other researchers linked an acute SARS-CoV-2 infection to HIV test reactivity and suggested but did not demonstrate the presence of cross-reactive Abs [15,17,18,19,20]. SARS-CoV-2 spike-specific IgM peaks 2–5 weeks after the onset of symptoms [31,32] but decays quickly. Indeed, these Abs have a half-life of 65–73 days as estimated using an exponential model [32,33]. An in-depth study found that 6 months after symptom onset, more than 77% of individuals lost their expression of spike-specific IgM [32]. Similarly, the HIV test interference waned over the course of more than 3 months and became undetectable within 8 months after the first documentation (10 months after SARS-CoV-2 infection) (Figure 1). Thus, we tested the presence of spike-specific IgM Abs as a possible cause of diagnostic test interference using direct ELISA. However, no IgM of that specificity was detected, thus excluding cross-reactive IgM Abs as an explanation (data not shown). Next, we explored whether SARS-CoV-2-specific Abs in patient 1 might cause HIV test reactivity, probing for SARS-CoV-2 spike/HIV-1 gp120 cross-reactivity. All samples collected from patient 1 contained SARS-CoV-2 spike-specific IgG. In December 2020, a qualitative confirmation (51.7 AU/mL with ≥15 AU/mL defined as IgG positive) was obtained using the Liaison SARS-CoV-2 S1/S2 IgG ELISA (DiaSorin). In March 2021 and after the first SARS-CoV-2 vaccination with Comirnaty (BioNTech) in August 2021, the quantitative titer of SARS-CoV-2 spike-specific IgG was determined with Liaison SARS-CoV-2 TrimericS IgG assay (DiaSorin) to be 120.6 BAU/mL and 1730 BAU/mL, respectively. While the positive signal in the confirmatory HIV tests waned over time and eventually fell below the detection threshold in August 2021 (Figure 1), SARS-CoV-2 spike-specific Abs were boosted by vaccination at that time point.

**Figure 3:**
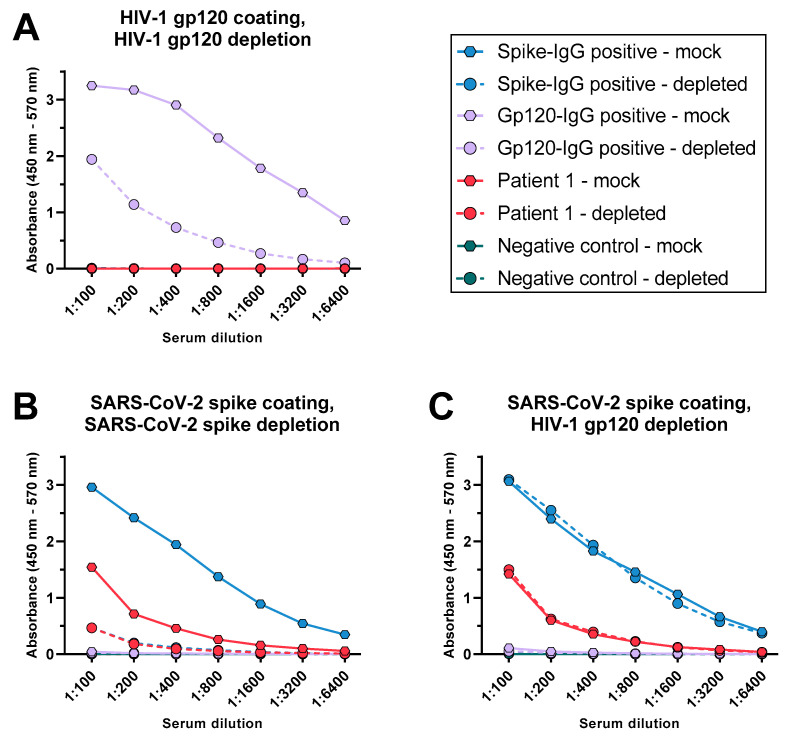
Test for the presence and cross-reactivity of SARS-CoV-2 spike-specific IgG in the serum of patient 1 collected in March 2021. Direct ELISAs were conducted measuring IgG binding to monomeric HIV-1 gp120 (**A**) or trimeric soluble SARS-CoV-2 spike (**B**,**C**). Serum samples were either mock treated or depleted for HIV-1 gp120 (**A**,**C**) or SARS-CoV-2 spike (**B**) using antigen-coupled magnetic beads. Depicted is the background subtracted mean signal. The SEM was calculated but is too small to be plotted with GraphPad Prism; n = 2.

The discrepancies in the kinetics of HIV test interference and SARS-CoV-2 spike-specific IgG might result from the further maturation of the spike-specific Ab response induced by vaccination and thus do not exclude the possible presence of cross-reactive Abs. Therefore, we set out to provide direct evidence for SARS-CoV-2 spike/HIV-1 gp120 cross-reactive Abs. For this purpose, the IgG Ab binding to HIV-1 gp120 and SARS-CoV-2 spike was determined with ELISA after the samples were either mock treated or depleted for Abs to SARS-CoV-2 spike or HIV-1 gp120 (Figure 3) using antigen-coupled magnetic beads. The signal for the HIV-1 gp120 or SARS-CoV-2 spike-specific IgG containing sera was depleted effectively (Figure 3A,B) in comparison to the negative control. The SARS-CoV-2 spike-specific IgG in the sera of patient 1 was equally efficiently depleted by spike-coated beads (Figure 3B), but neither binding to gp120 (Figure 3A) nor a loss of spike reactivity upon depletion treatment with gp120 was observed (Figure 3C). Based on these findings, we conclude that no cross-reactive IgGs were present in the sample of patient 1 that recognize both SARS-CoV-2 spike and HIV-1 gp120.

## 5. Discussion

Our study adds to the evidence already compiled by others suggesting an association between acute SARS-CoV-2 infection and false-reactive results in HIV diagnostic tests. In contrast to other studies, patient 1 tested positive not only in a screening test but also in a confirmatory test. German/Austrian diagnostic guidelines require a positive screening and confirmatory test for an HIV diagnosis. Subsequently, a suspected HIV infection needs to be confirmed by repeating the assays using an independently collected sample [1]. Thus, patient 1 only received a suspected but not an official HIV diagnosis. Regardless, we cannot exclude the general possibility of a false HIV diagnosis due to SARS-CoV-2 infection. For patient 1, the screening assays used for the analysis of the samples in December 2020 varied between institutions and produced different results within only 12 days. These differences might originate from the quick resolution of the assay interference, apparently capable of impacting multiple screening systems [15,16,17,19,20,21] or could be due to variable assay setups and sensitivities. While the screening assays for patient 1 were nonreactive in March 2021, the assay interference remained detectable with LIA and thus lasted for at least 3 months (Figure 1). In our cohort study, we identified no other individuals showing HIV test interference (Table 1), suggesting HIV test interference, if caused by SARS-CoV-2 infection, to be not very frequent and/or short-lived. This is in accordance with the work of other groups who observed 1.4–1.7% more false-reactive HIV screening tests in individuals with past SARS-CoV-2 infection [15,16]. However, considering the waning of observed assay interference over time, the relevance of the sample collection time point post SARS-CoV-2 infection for HIV diagnostics should be investigated further.

After assessing the potential influence of a variety of common reasons for assay interferences, we tested the hypothesis that SARS-CoV-2-induced HIV cross-reactive Abs were responsible for the observation. The absence of spike-specific IgM (data not shown) and the lack of binding of spike-specific IgG to HIV-1 gp120 (Figure 3), disproved this hypothesis. Based on these findings, we conclude that in our case, cross-reactive Abs were not the cause of the diagnostic test interference. Nevertheless, there might still be a link between an acute SARS-CoV-2 infection and false-positive HIV tests. Several pathogens have been shown to induce nonspecific polyclonal B cell responses, including active EBV infection [34]. While we detected no Ab responses indicating EBV replication in patient 1, we cannot exclude the induction of nonspecific B cell responses by other pathogens. Indeed, studies showed that SARS-CoV-2 stimulates a polyclonal autoreactive B-cell response [35,36]. In hospitalized COVID-19 patients, this included the induction of rheumatoid factor [37], a group of autoantibodies capable of cross-linking human and animal Abs and interfering with diagnostic assays including HIV screening tests [38,39]. Thus, SARS-CoV-2 infection in patient 1 might have induced a transient autoreactive B-cell response resulting in the production of Abs capable of cross-linking HIV diagnostic test components.

The work shown here is limited by the choice of antigens, namely SARS-CoV-2 spike and HIV-1 gp120. Besides SARS-CoV-2 spike, N protein is another strong stimulator of the humoral immune system [33]; however, no study thus far has suggested a cross-reactivity between SARS-CoV-2 N protein and HIV Env. In the HIV LIA, reactive bands for both gp120 and gp41 were observed, and both protein reactivities waned and disappeared together. Thus, while a nonspecific test interference seems rather likely, theoretically, a combination of nonspecific test interference with gp41 cross-reactive Abs is another possible explanation. An additional limitation was the small size of the cohort of post-COVID individuals, which did not allow for an estimation of the test interference frequency, as well as the late sampling time point of 9 months post SARS-CoV-2 infection.

In summary, SARS-CoV-2-associated HIV test interference is a short-lived and/or rare process, which can interfere with both HIV screening and confirmatory tests. SARS-CoV-2 spike/HIV-1 Env cross-reactive Abs did not underlie this process in our patient, and other possible causes, including virus-induced autoantibodies, should be examined. Finally, in the case of unexpected HIV test results, physicians should include questions about the SARS-CoV-2-related medical history into the conversation with their patients.

## Figures and Tables

**Table 1:** Description and test results for study participants receiving treatment for post-COVID.

**Characteristics**	**Total**	**Female**	**Male**
Number of patients, n (%)	65 (100)	42 (64.6)	23 (35.4)
Age in years, median (IQR)	51 (19)	50.5 (18)	52 (20.5)
Time post SARS-CoV-2 diagnosis, median months (IQR)	9 (5) *	9 (4.3)	9 (4.8)
Vaccination status			
Nonvaccinated, n (%)	4 (6.2)	2 (4.8)	2 (8.7)
Vaccinated, n (%)	53 (81.5)	32 (76.1)	21 (91.3)
Vaccination status unknown, n (%)	8 (12.3)	8 (19)	0 (0)
**Laboratory investigation**	**Total**	**Female**	**Male**
SARS-CoV-2 spike-specific IgG Liaison SARS-CoV-2 TrimericS IgG assay (DiaSorin)			
Positive, n (%)	63 (96.9)	40 (95.2)	23 (100)
Negative, n (%)	2 (3.1)	2 (4.8)	0 (0)
SARS-CoV-2 N protein-specific IgG SARS-CoV-2 IgG assay (Abbott)			
Positive, n (%)	27 (41.5)	16 (38.1)	11 (47.8)
Borderline, n (%)	9 (13.8)	7 (16.7)	2 (8.7)
Negative, n (%)	29 (44.6)	19 (45.2)	10 (43.5)
HIV-1/2 screening assay Elecsys HIV combi PT (Roche)			
Positive, n (%)	0 (0)	0 (0)	0 (0)
Negative, n (%)	65 (100)	42 (100)	23 (100)
Architect HIV Ag/Ab Combo test (Abbott)			
Positive, n (%)	0 (0)	0 (0)	0 (0)
Negative, n (%)	65 (100)	42 (100)	23 (100)
HIV-1/2 confirmatory immunoblot INNO-LIA HIV I/II Score (Fujirebio)			
Positive, n (%)	0 (0)	0 (0)	0 (0)
Negative, n (%)	65 (100)	42 (100)	23 (100)

* Three participants (1 male, 2 female) were excluded from the analysis due to insufficient information.

## Data Availability

The data presented in this study are available on request from the corresponding authors.
